# Six weeks of whole-body vibration improves fine motor accuracy, functional mobility and quality of life in people with multiple sclerosis

**DOI:** 10.1371/journal.pone.0270698

**Published:** 2022-07-11

**Authors:** Anne Krause, Kyungsoo Lee, Daniel König, Michael Faist, Kathrin Freyler, Albert Gollhofer, Ramona Ritzmann

**Affiliations:** 1 Department of Sport Science, University of Freiburg, Freiburg, Germany; 2 Institute of Training and Computer Science, German Sport University Cologne, Cologne, Germany; 3 Department of Sport Science and the Department of Nutritional Sciences, University of Vienna, Vienna, Austria; 4 Department of Neurology and Clinical Neurophysiology, University Hospital Freiburg, Freiburg, Germany; 5 Department of Biomechanics, Praxisklinik Rennbahn, Muttenz, Switzerland; Mugla Sitki Kocman Universitesi, TURKEY

## Abstract

People with multiple sclerosis (MS) suffer from sensorimotor deficits with the distal extremities being more severely affected than proximal ones. Whole-body vibration (WBV) training is known to enhance voluntary activation and coordination in healthy people. However, evidence about beneficial effects of WBV in MS patients is scarce. The current study aimed to investigate if six weeks of WBV enhances motor function in the ankle joint, coordination and quality of life in patients suffering from severe MS. In a longitudinal design, changes in motor function and quality of life were assessed before and after a 6-week control period without a training (CON) and a 6-week WBV training (2-3x/week) in 15 patients (53 ±10 years) with advanced MS (EDSS 3–6.5). Before CON (t_0_), after CON (t_1_) and after WBV(t_2_), outcome measures included (1) active range of motion (aROM) and (2) motor accuracy at the ankle joint, (3) functional mobility (Timed “Up & Go” test with preferred and non-preferred turns) and (4) physical and psychological impact of MS (MSIS-29 questionnaire). For (1) and (2), the stronger (SL) and the weaker leg (WL) were compared. After WBV, aROM (1) did not change (SL p = 0.26, WL p = 0.10), but was diminished after CON (SL -10% p = 0.06, WL -14% p = 0.03) with significant group differences (Δgroup WL p = 0.02). Motor accuracy in SL (2) was improved during dorsal flexion after WBV (p = 0.01, Δgroup p = 0.04) and deteriorated during plantar flexion after CON (p = 0.01, Δgroup p = 0.04). Additionally, participants (3) improved their functional mobility at the preferred turn (p = 0.04) and (4) ranked their quality of life higher solely after WBV (p = 0.05), without any differences between groups. However, values correlated significantly between angular precision and aROM as well as functional mobility. No further changes occurred. The results point towards an interception of degenerating mono-articular mobility and improvement of accuracy in the ankle joint. The motor effects after WBV are in line with enhanced perception of quality of life after six weeks which is why WBV could be a stimulus to enable greater overall autonomy in MS patients.

## Introduction

Multiple sclerosis (MS) is a progressive, chronic disease of the central nervous system (CNS). MS disrupts the information transmission within the CNS which leads to sensory [1] and motor dysfunctions [2] with the consequence of increased fragility, loss of autonomy and an impaired quality of life [3–5]. MS symptoms are asymmetric and affect body sides differently [4, 5]. In addition to the disease itself, physical inactivity increases the propensity for a reduced motor and cardiorespiratory performance [3], activities of daily living [4], and wellbeing [3]. Thereby, a significant worsening of MS symptoms is associated with lower physical activity levels [5] whereas MS-induced impairments can be mitigated in individuals engaged in exercise programs [4]. However, common exercises require a safe bipedal stance and locomotor mobility [5] which is often not existent for patients with an advanced disease stage of Expanded Disability Status Scale scores (EDSS, [6]) >4 [3].

On the basis of neuroprotective effects of physical activity, including an increased release of neurotrophic factors [7] and an inhibited demyelination [8], moderate physical exercise could break the disease-induced cycle of inactivity and immobility [9]. Efficient and easy-to-apply in everyday life [10], training with whole-body vibration (WBV) fulfills the precondition of a safe exercise mode while avoiding thermogenesis in patients with MS [11]. Mechanical oscillations are transmitted through the entire body as an indirect stimulus to act on human neuromuscular structures [12]. WBV operates via spinal Ia afferent reflex responses elicited by an alternating stretching and shortening of the affected musculature [13]. It is either executed in isometric conditions or in combination with dynamic types of movement in populations affected by neuro-degenerative diseases [12]. The feasibility and efficacy of WBV is independent of the subjects’ movement ability, health and mental status [14]. Therefore, the neuro-rehabilitative application of vibration has emerged as a valuable tool with a high compliance in MS patients [15] as there are no decisive motor or cognitive prerequisites [14].

With a total of 12 studies there is just minor level of evidence about the long-term effect of WBV on motor performance, functional mobility and quality of life in MS populations. Meta-analysis revealed an increased muscle strength [16] whereas no benefit of WBV on posture control [15] and functional mobility have been reported when compared with outcomes in the control groups [17]. Evidence about the effects of WBV in fine motor coordination, spasm, improving gait [18], wellbeing or quality of life [17] was characterized to be poor to moderate and not fully understood. Most of the included trials had no control group, short intervention periods (<6 weeks) and were conducted in mobile MS populations with EDSS < 3 without consideration of symptomatic asymmetries with respect to laterality and the stronger and weaker body sides [4, 5]. Likewise, effects on the distal leg segment have not yet been assessed although the gait has been related to severe advances in gait dysfunction [19] and a drop foot when the EDSS score is beyond 3.5 [20]. Therefore, an evaluation of mobility in the ankle joint coupled with a decidedly clustered quality of life assessment is necessary to conclude the efficiency of WBV as a countermeasure to antagonize physical deconditioning in patients with MS.

Based on the above-mentioned rationale and lack of knowledge, the aim of the current study was to investigate, if (a) mono-articular mobility and (b) accuracy is enhanced in MS patients after a 6-week period of WBV. Further, the aim was to establish if these improvements are related to (c) enhanced functional mobility during gait and (d) the quality of life or autonomy in everyday life. Patients with advanced MS (EDSS >3) were of particular interest for the study. As distal body regions are commonly highly affected by MS, a test battery was used that entails an assessment of fine motor coordination and accuracy in the ankle joint including mono-articular plantar and dorsal flexion with respect of laterality of the stronger and weaker leg [4, 5], gross motor control by means of functional mobility in the Timed “Up & Go” test (TUG) test [21] and quality of life by means of physical and psychological rating scale Multiple Sclerosis Impact Scale (MSIS-29) [22]. Based on the results of an a priori pilot study, it is hypothesized that WBV would increase fine motor coordination and active range of motion (aROM) in the ankle joint and would improve mobility in TUG and MSIS scores.

## Materials and methods

### Design

Over a period of 3 months, the effects of a 6-week WBV training were tested using a repeated measures longitudinal design. Prior to WBV intervention, a 6-week control period (CON) with no intervention and continuation of everyday life activities was used to evaluate the participants’ health status and disease-induced decline in motor control. All participants were assigned to CON and WBV. Before (t_0_) and after CON (t_1_) as well as after WBV (t_2_), a random test battery with four assessments was executed. The study design was approved by the ethics committee of the University of Freiburg (189/15). The study was registered at the German Clinical Trials Register with the ID DRKS00011892. All participants gave written informed consent to the experimental procedure. Data were assessed at the University of Freiburg.

### Screening and participant selection

The sample size calculation was based on the effect sizes observed in a pilot experiment with n = 5. The a priori power analysis (G*Power V 3.1.9.2) revealed a sample size of N = 15 for non-parametric Wilcoxon Test and an effect size of d = 0.3, a power = 0.85 and an α < 0.05 for the parameter aROM, TUG time and the MSIS-29 score.

**Fig 1:**
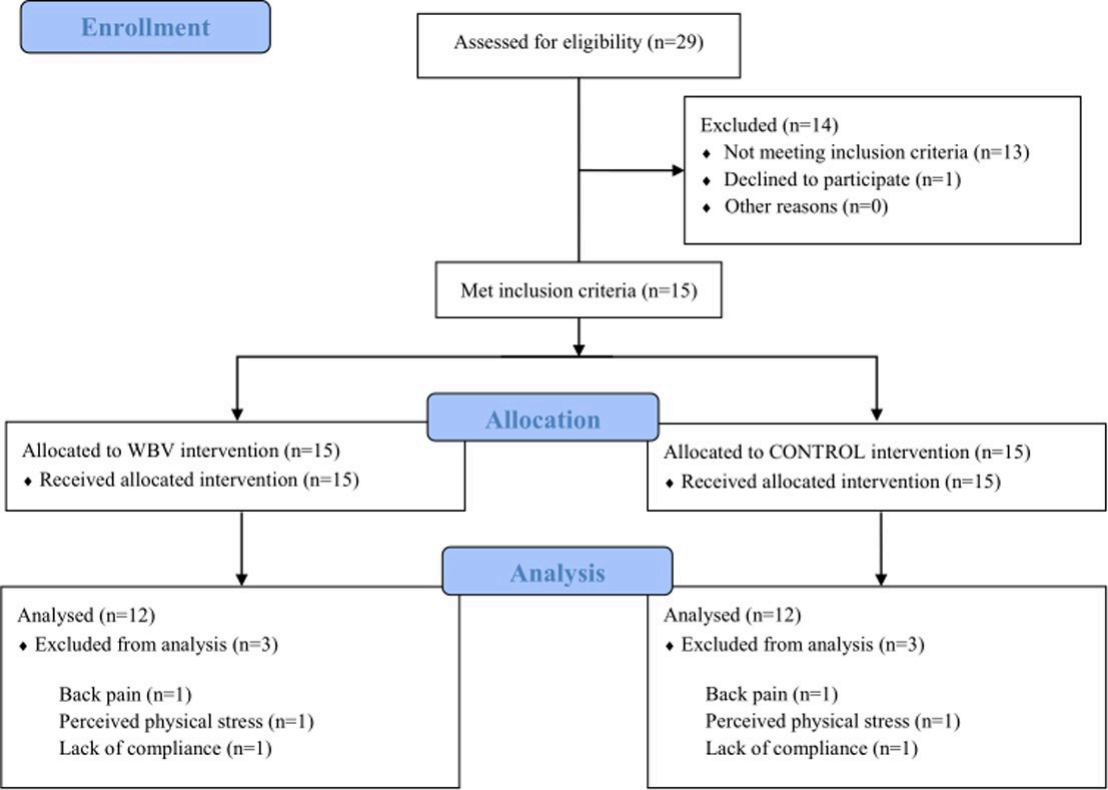
Flow chart diagram.

Recruitment took place between November 2015 and August 2016 using a newspaper advertising. From 29 volunteers who underwent medical investigation, a total of 15 met the inclusion criteria (Fig 1). Those comprised a diagnosis of MS with lower limbs being affected; stable medical condition and EDSS values of 3–6.5 to include participants who were still able to walk with or without walking aids [6]. Patients with relapses in the preceding three months, acute injuries, other chronic diseases and/ or uncontrolled hyper- or hypotension were excluded. Documentation during the clinical visit involved the patient characteristic (age, height, weight, EDSS score), medical assessment of the stronger and weaker leg [1] and the preferred direction during gait turn [21]. Patient characteristic is presented in Table 1.

**Table 1:** Characteristics of participants.

	Values
**Sex (f/m)**	11/4
**Age (years)**	53 ± 4
**EDSS**	5.5 (4–6)
**Height (cm)**	170 ± 9
**Weight (kg)**	73 ± 2

The trial started in August 2016 and ended in December 2016 as soon as the last participant finished the last assessment; the authors conducted and supervised the intervention.

### Assessments

**Fig 2:**
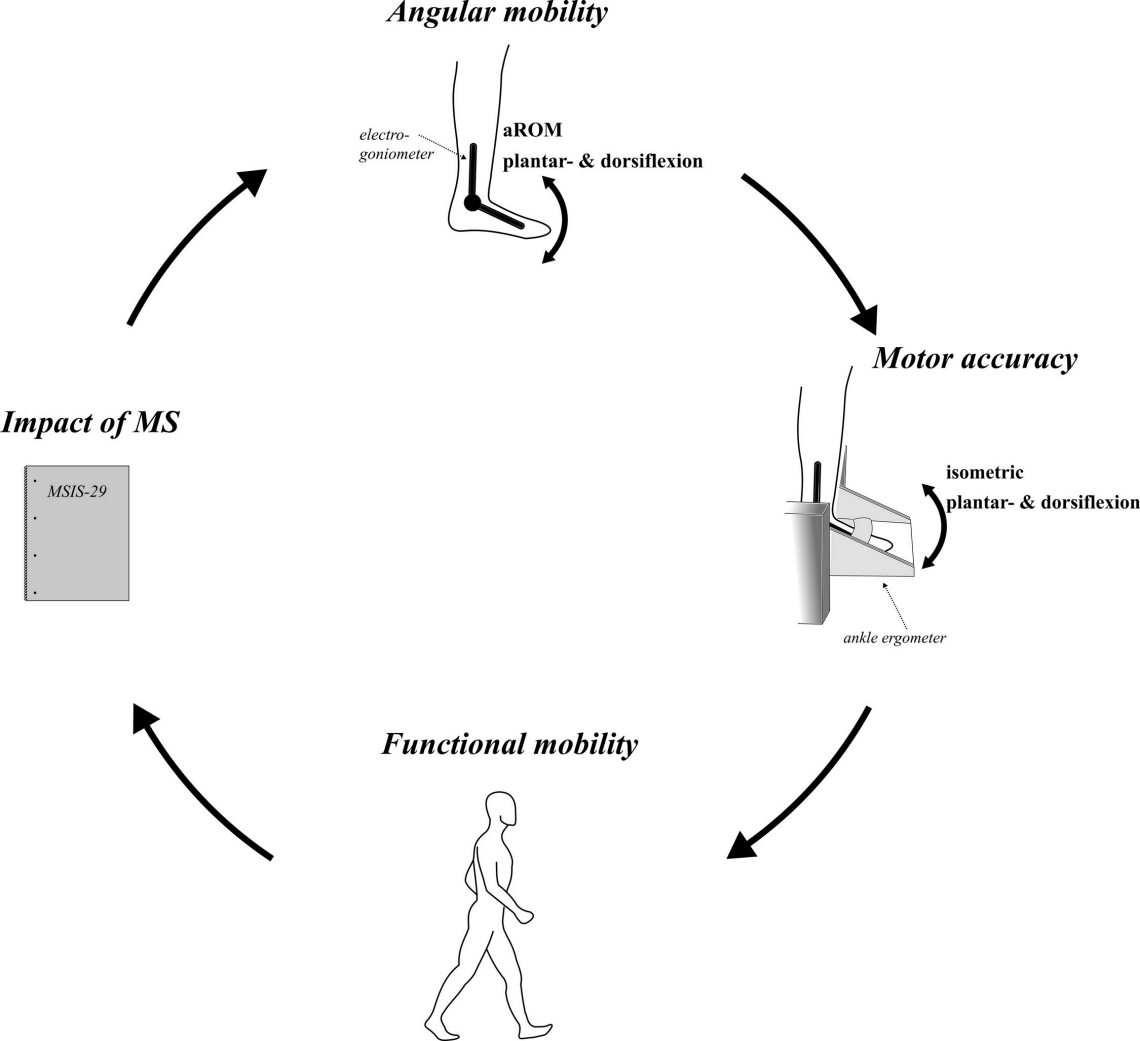
Overview of the applied assessments. The assessments included (1) angular mobility by means of aROM measures in the ankle joint using electro-goniometry and (2) motor accuracy by means of plantarflexion and dorsal extension with biofeedback. Additionally, (3) functional mobility was assessed by means of the Timed “Up & Go” test (TUG) and (4) impact of MS with the Multiple Sclerosis Impact Scale (MSIS-29).

Three assessment time points were carried out at the laboratory of the University of Freiburg: one before and one after a six-week control period (CON) and one after the following six-week of WBV training. CON was set before WBV to assess the degeneration status of each participant. Therefore, participants continued their everyday life activities including physio and exercise therapy, mobilization and medical treatment. Prior to CON, after CON and after WBV, four assessments were conducted in a randomized order to analyze (1) angular mobility, (2) motor accuracy, (3) functional mobility, and (4) the impact of MS during everyday life activities (Fig 2).

To assess angular mobility (1), participants were seated, maximally bended and extended their ankle joint from full extension to full flexion [23]. Active range of motion at the ankle joint (aROM) was measured with monoaxial goniometers (Biometrics®, Gwent, UK). Goniometers were placed with their center of rotation over the rotational axis of ankle joint (malleolus lateralis). The two parts of the goniometers were aligned with the joint axes pointing towards the fifth metatarsal and longitudinal axis of the shank, respectively. Trials were repeated three times.

**Fig 3:**
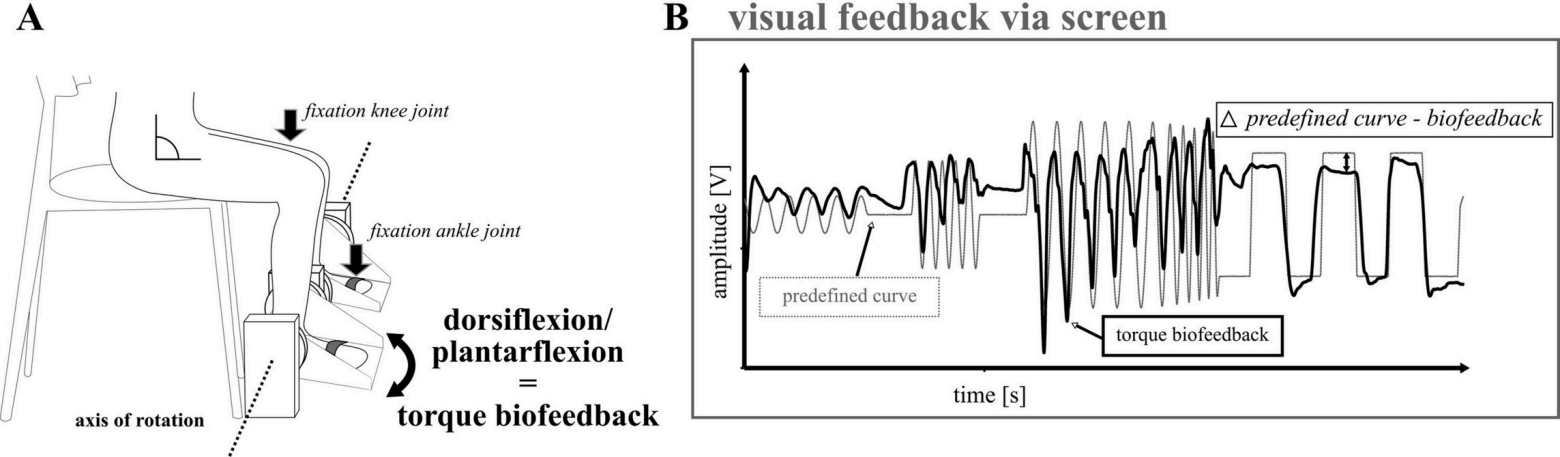
Assessment setup for motor accuracy. Participants sat upright in a chair and were asked to flex and extend their ankle joint (A). Fixations were provided at the knee and ankle; the ankle joint was in line with the rotation axis of the ergometer. Ankle joint position was visualized for plantarflexion and dorsal extension (B) as biofeedback. Participants were asked to replicate the predefined (grey) curve with as accurately as possible (black). Errors were calculated by means of differences between the predefined curve and the position signal in Δ°.

Motor accuracy (2) was assessed in the ankle joint in a reproduction task of angular movement [26]. Participants sat in a custom-build ankle-ergometer (Fig 2); the ankle joint was in line with the rotation axis. The knee and hip were fixed at 90° flexion. In front, visual feedback of the ankle joint position was illustrated on a screen ranging from 50° plantarflexion to 30° dorsal extension. Participants were instructed to trace a stochastically predefined sinusoidal-rectangular curve with an appropriate and accurate ankle joint movement as precisely as possible (Fig 3). A total of three trials were recorded with a duration of 30 s and a frequency of 200 Hz. To exclude habituation effects, participants practiced the task for 3min prior to the data recording.

Changes in functional mobility (3) were assessed with the TUG [24]. The direction of turn was given by the documentation of the medical visit. Participants started with the preferred direction (TUG_preferred_) followed by the non-preferred direction (TUG_non-preferred_). With standardized instructions, time to complete as outcome measure has been described to correlate strongly to the level of functional mobility [21]. (TUG_preferred_ and TUG_non-preferred_ were repeated twice. Participants conducted one trial for familiarization.

In addition, the impact of MS during everyday life activities (4) was assessed with the reliable and valid questionnaire MSIS-29 [22]. With a self-report five point Likert scale (“not at all, a little, moderately, quite a bit, extremely”), participants rate in 29 items (20 physical, 9 psychological items) their perceived impact of MS. It took about 10 min to complete with total score ranging from 29 (lowest degree of disability) up to 145 (greatest degree of disability) [22].

### Intervention

The intervention protocol is described and graphically illustrated in Krause et al. [25]. All participants underwent a 6-week control period, followed by an intervention period of 6-week WBV. Throughout the study, participants were asked to continue to carry out their daily activities. WBV was conducted on a side-alternating sinusoidal WBV platform (Galileo Sport, Novotec Medical GmbH, Pforzheim, Germany). Participants trained either at home (n = 7) or at the laboratory (n = 5). Three participants dropped out during the intervention due to intolerance of the training (n = 1 back pain, n = 1 perceived physical stress) and lack of compliance (n = 1). In any case, participants trained two to three times a week, with a minimum regeneration period of 24h, resulting in 12–18 training sessions in total.

The training was standardized throughout the whole intervention period. To provide an individualized trainings stimulus, training intensity was weekly defined based on the level of perceived exertion with the validated BORG scale [26]. The standardized protocol included the following characteristics. One session lasted 20-30min and included a warm-up part (i) of 3x3 static exercises with a duration of 21±3s (min. 20s and max. 30s) and frequencies of 17±1Hz (min.16Hz and max.19Hz). Training part (ii) consisted of 3x3 dynamic exercises with longer durations of 50±5s (min. 40s and max. 75s) and frequencies of 25±1Hz (min. 23Hz and max. 30Hz). Recovery periods between exercises were kept constant with 60s [27]. As described elsewhere, basic position was with feet hip-width apart (amplitude 1.9±0.2mm, peak-to-peak displacement 3.7±0.74mm), heels slightly lifted, knees slightly bent and hands lying on the supporting rails of the vibration device in case of a loss of balance (cf. S1 Fig) [25].

Perceived exertion was assessed with values of the BORG scale (6–20). Values ranged between 12 (“somewhat hard”)– 16 (“hard”). If values were >16 or participants felt (neurological) fatigue, training was reduced for the next session. The modifications of the individualized intensity were based on the following characteristics: Load was increased gradually by means of (1) duration of the exercise (+5s, max.75s), (2) frequency (max.30 Hz) and (3) complexity of exercise (e.g. lowering chair height at chair-rising task, adding load during squats, no uses of handrails). Training was documented in a diary daily, supervised weekly (including home visits) and adjusted by the authors throughout the study period.

### Data analysis

For assessments (1) to (3), data are presented in mean values ± standard deviations. For assessment (4), median values were calculated due to the ordinal scale. For presentation purpose either raw data (absolute values) or relative values given by differences (t_0_-t_1_ or t_2_-t_1_) and quotients (t_0_/t_1_ or t_2_/t_1_) were used.

For angular mobility (1), goniometers at the ankle joint were set to zero with an angle of 90° between foot and shank. To calculate maximum aROM among three trials, the minimum angle was subtracted from maximum angle for the stronger and weaker leg, respectively.

For motor accuracy (2), differences (Δ°) between the predefined sinusoidal-rectangular curve and the participants traces for plantarflexion and dorsal extension were calculated, respectively. Δ° refers to the error; the greater the error the smaller the motor accuracy. The total Δ° was averaged among three trials for the stronger and weaker leg, respectively.

For functional mobility (3), time to finish the test was divided up into preferred turn (first choice, TUG_preferred_) and non-preferred turn (second choice, TUG_non-preferred_).

For impact of MS during everyday life (4), item scores were added for the physical (20 items) and for the psychological scale (9 items) and summarized as a total score (all 29 items). Each scale was scored by summing. The whole data analysis was conducted and controlled by the authors of the study.

### Statistics

All statistical calculations were conducted with the statistics software SPSS 20.0 (SPSS, Inc., Chicago, IL, USA). Normal distribution was assessed with the Kolmogorov-Smirnov Test. Based on the results, either one-tailed Student’s t-Tests or Wilcoxon Tests were calculated, including Benjamini-Hochberg corrections for multiple testing. For impact of MS during everyday life (4), solely non-parametric tests due to the ordinal scale of the items were used. Group effects were assessed with one-sided Wilcoxon-Test for repeated measures. For all values, effects sizes between time points were calculated with Cohen’s d with the following reference values: small effects sizes for 0.2 < *d* < 0.5, medium effect sizes for 0.5 < *d* < 0.8 and large effects sizes for *d* > 0.8 [28].

One-tailed Spearman correlation coefficients were calculated for the respective percentage changes obtained in t_2_ compared to t_1_ to determine the strength of linear relations between the WBV-induced adaptations in active aROM, fine motor accuracy, TUG or MSIS-29 sores.

## Results

For angular mobility (1), aROM in the ankle joint did not change after six weeks of WBV for the weaker (p = 0.10) nor stronger leg (p = 0.26). However, this is in contrast to a significantly diminished aROM by -14% after CON for the weaker leg (p = 0.03, *d* = 0.76) with significant differences between CON and WBV (p = 0.02). No significant differences within and between groups but small effect sizes (*d* = 0.24) were measured for aROM in the stronger leg. (cf. Table 2).

**Table 2:** Results of angular mobility and motor accuracy for the active range of motion (top) and motor precision task (bottom) of the ankle joint.

				ΔCON		ΔWBV		Δgroups
	t_0_	t_1_	t_2_	p	*d*	p	*d*	p
Ankle_SL_ [°]	52.9 ±23.4	47.4 ± 24.2	57.9 ±30.3	0.06	*0*.*24*	0.26	*0*.*40*	0.12
	[37.16; 68.66]	[31.09; 63.61]	[37.52; 78.28]					
Ankle_WL_ [°]	**56.1 ±11.0**	48.4 ± 10.3	53.5 ±9.7	**0.03**	*0*.*76*	0.10	*0*.*54*	**0.02**
	**[48.70; 63.54]**	[41.48; 55.28]	[46.99; 60.08]					
Error PF_SL_ [a.U.]	**0.040 ±0.011**	0.047 ±0.013	0.040 ±0.009	**0.01**	*0*.*56*	0.08	*0*.*63*	**0.01**
	[0.03; 0.05]	[0.04; 0.05]	[0.03; 0.05]					
Error DF_SL_ [a.U.]	0.056 ±0.022	0.055 ±0.015	**0.047 ±0.011**	0.90	*0*.*02*	**0.01**	*0*.*67*	**0.04**
	[0.04; 0.07]	[0.05; 0.06]	[0.04; 0.05]					
Error PF_WL_[a.U.]	0.041 ±0.011	0.040 ±0.010	0.036 ±0.007	0.79	*0*.*08*	0.06	*0*.*48*	0.29
	[0.03; 0.05]	[0.03; 0.05]	[0.03; 0.04]					
Error DF_WL_[a.U.]	0.049 ±0.018	0.050 ±0.016	0.051 ±0.013	0.91	*0*.*04*	0.40	*0*.*06*	0.41
	[0.04; 0.06]	*[0*.*04; 0*.*06]*	[0.04; 0.06]					

Listed are all mean values ± standard deviations and 95% CI in square brackets before (t_0_) and after six weeks of no intervention (t_1_, ΔCON), as well as after six weeks of whole-body vibration intervention (t_2_, ΔWBV). Significant results are highlighted in bold letters. Cohen’s *d* is presented with small (0.2 < *d* < 0.5), medium (0.5 < *d* < 0.8) and large effects sizes (*d* > 0.8). Note that motor accuracy increases with reduced error values. P values for differences between groups are listed in the last column as *Δ groups*.

CON = Control period; WBV = Whole-body vibration intervention; PF = Plantar flexion; DF = Dorsal flexion; SL = Stronger leg; WL = Weaker leg; Δ = Differences

**Fig 4:**
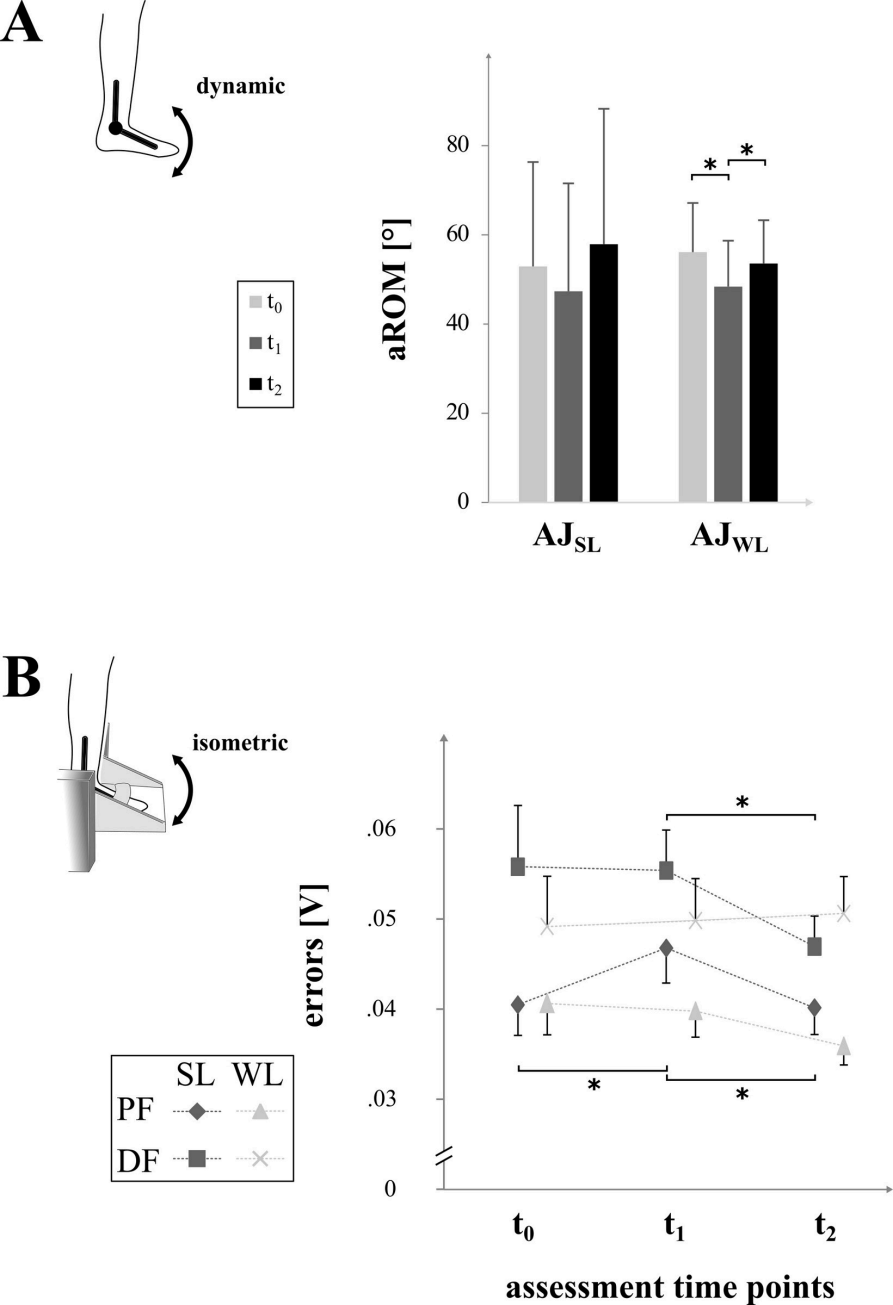
Angular mobility and motor accuracy. The graphs illustrate changes of mono-articular mobility (A) and motor accuracy (B) prior (t_0_), after six weeks of no intervention (t_1_) as well as after six weeks of WBV (t_2_). “A” shows significant changes with an asterisk of active range of motion (aROM) in [°] for the ankle joint of the stronger leg (AJ_SL_) and weaker leg (AJ_WL_). “B” illustrates errors (in [V]) during fine motor tasks for the plantar flexion (PF) and dorsal flexion (DF) with the stronger leg (SL, dark grey) and the weaker leg (WL, light grey).

For motor accuracy (2), statistical analysis revealed an increase in motor accuracy of the ankle joint after WBV in the stronger leg during dorsiflexion (-15%, p = 0.01, *d* = 0.67) and plantar flexion (-14%, p = 0.08, *d* = 0.63) with medium effect sizes and significant differences between groups (p = 0.04 & p = 0.01, respectively). In contrast, after CON, motor accuracy was reduced for plantar flexion (+13%, p = 0.01, *d* = 0.56, cf. Figs 3 and 4), but not dorsiflexion (p = 0.90, *d* = 0.02). In the weaker leg, no significant changes occurred with trivial effect sizes for dorsiflexion (0.04–0.06) and trivial to small effect sizes for plantarflexion (0.08–0.48, cf. Table 2).

For functional mobility (3), the test was divided up into turning preference. Participants needed significantly less time to complete the task after WBV during TUG_preferred_ (-10%, p = 0.04, *d* = 0.31), but not for TUG_non-preferred_ (p = 0.32, *d* = 0.15). Neither significant differences were observed after CON for TUG_preferred_ (p = 0.19) nor for TUG_non-preferred_ (p = 0.13) with no between-group differences and trivial to small effect sizes (0.15–0.20, cf. Table 3).

**Table 3:** Results of functional mobility and impact of MS during everyday life for the Timed “Up & Go” test (top) and for the MSIS-29 questionnaire scores (bottom).

				ΔCON		ΔWBV		Δgroups
	t_0_	t_1_	t_2_	p	*d*	p	*d*	p
TUG [s]	14.6 ±6.3	13.6 ± 5.5	12.5 ±4.4	0.09	*0*.*18*	0.34	*0*.*23*	0.48
	[10.06; 19.10]	[9.64; 17.51]	[9.35; 15.62]					
TUG_preferred_ [s]	15.1 ±6.7	13.9 ± 6.0	**12.4 ±3.9**	0.19	*0*.*20*	**0.04**	*0*.*31*	0.44
	[10.33; 19.86]	[9.60; 18.14]	**[9.59; 15.19]**					
TUG_non-preferred_ [s]	14.1 ±6.0	13.3 ± 5.2	12.6 ±4.9	0.13	*0*.*15*	0.32	*0*.*15*	0.40
	[9.77; 18.36]	[9.57; 16.99]	[9.08; 16.08]					
MSIS-29 total score [a.U.]	2.56	*2*.*33*	**2.17**	0.23	*0*.*42*	**0.05**	*0*.*61*	0.19
MSIS-29 Phy. score [a.U.]	2.93	*2*.*83*	**2.51**	0.18	*0*.*28*	**0.04**	*0*.*55*	0.13
MSIS-29 Psy. score [a.U.]	2.50	*2*.*03*	1.72	0.44	*0*.*36*	0.11	*0*.*42*	0.44

Listed are all mean values ± standard deviations 95% CI in square brackets for Timed “Up & Go” and median values for MSIS-29 before (t_0_) and after six weeks of no intervention (t_1_, CON), as well as after six weeks of whole-body vibration intervention (t_2_, WBV). Significant results are listed with p < 0.05 in bold letters. Cohen’s *d* is presented with small (0.2 < *d* < 0.5), medium (0.5 < *d* < 0.8) and large effects sizes (*d* > 0.8). P values for differences between groups are listed in the last column as *Δ groups*.

CON = Control period; WBV = Whole-body vibration intervention; TUG = Timed “Up & Go” test; MSIS-29 = Multiple Sclerosis Impact Scale 29; Phy. = Physical; Psy. = Psychological

For impact of MS during everyday life (4), changes in MSIS-29 scores revealed that the perceived impact of MS was diminished by -13% in total (p = 0.05, *d* = 0.61), with reduced values of -13% for the physical impact score after WBV (p = 0.04, *d* = 0.55). No significant changes were measured after CON (p = 0.23, p = 0.18, respectively) with small effect sizes (0.28–0.42). The psychological impact score did not change after CON (p = 0.44, *d* = 0.36) nor WBV (p = 0.11, *d* = 0.42). No differences between groups were observed (total p = 0.19, physiological impact p = 0.13, psychological impact p = 0.44, cf. Table 3).

After WBV, the increase in motor accuracy in the ankle joint of the stronger leg correlated negatively with time to complete TUG_preferred_ (r_s_(10) = -0.657, p<0.05). Likewise, positive correlations were observed for the increase in aROM of the ankle joint of the stronger leg and motor accuracy of dorsal flexion (r_s_(11) = 0.566, p<0.05) and plantar flexion (r_s_(11) = 0.560, p<0.05). TUG_preferred_ correlated positivity with MSIS-29 scores (r_s_(10) = 0.594, p<0.05, cf. Table 3).

## Discussion

This study permits a novel perspective on how WBV affects fine motor coordination with an emphasis on the lower extremities, functional mobility and quality of life in patients with MS. The disease-tailored WBV exercise was effective in (a) preventing deficits in mono-articular mobility and (b) improving motor accuracy. Improvements in motor mobility and accuracy of the stronger leg correlated with an enhanced functional mobility and–indirectly–MSIS-scores, respectively. These outcomes provide evidence of the interrelationship between distal joint control, gross motor function and psychometrical patient-reported outcome of the MS disease.

### From mono-articular to complex movements

The emphasis was on the effect of WBV on angular mobility in the ankle joint and the effect of distal joint mobility on complex motion involving the entire body [29]. In patients with MS, irregular gait characteristics such as a reduced joint motion [19] or a drop foot [20] are described as major symptoms. The vibration stimulus could partly impede those degenerations. Functionally, those mono-articular adaptations become inalienable during everyday life movements: While MS-associated gait can be characterized as slow, instable and variable [19], motor performance may be improved by enhancing mobility and accuracy around the ankle joint. It is noteworthy that the sidedness plays a key role as stronger leg benefits most from the WBV training as indicated by the results of the correlations. This is particularly surprising and opens the fore to new research questions concerning motor asymmetries and the adaptability to training with reference to the disease MS.

The interrelationship between mono-articular to complex movements is also supported by significant correlations between motor accuracy and TUG. Nonetheless, TUG time which is a reliable measure to assess functional mobility, was only reduced if participants could take a turn in their preferred direction with effects that are smaller than reported clinically relevant changes of >23%. This is in line with previous results [17], which described either no [18] or just small improvements over time in functional mobility following a WBV training [16]. It can be supposed that the time to accomplish TUG is highly dependent on the quality of gait and posture control [30]. It is further hypothesized that turning to the non-preferred side might be characterized by greater postural instability and insecurity. However, as there are no improvements of WBV on posture control in MS patients [17], it can be assumed that current functional adaptations do not become evident in more dynamic–postural-challenging–movements as well.

### Underlining mechanism

Interestingly, aROM correlates with motor accuracy. This relationship might be explained by greater access of voluntary motor control during isometric force tasks. Although experimental evidence for the neuronal mechanisms underlying functional adaptations in MS patients is still lacking, there is at least clear evidence from healthy populations: Vibration is known to reduce spinal excitability [31] while corticospinal pathways are facilitated [32]. Therefore, a reflex inhibition during an active muscle stretch (as it is induced in the motor accuracy protocol) might have enabled a greater angular mobility which can be of advantage when the motor cortex commands simple voluntary motion [29]. This is in line with observations of a greater ankle torque during longer muscle lengths after the acute application of WBV [33] and might emphasize a close relationship between mono-articular mobility and motor accuracy which becomes evident in fine motor performance.

Despite the laboratory setting and partly insignificant group differences for the complex movements involving the entire body, participants reported a reduced impact of their disease in regard to their quality of life, especially regarding physiological tasks. Informal reports such as “walking without walking aids”, “smooth gait pattern” and “enhanced mobility during everyday life movements” were described by the participants. Thus, the improvements of mono-articular mobility to functional performance became evident in everyday life situations, which resemble the most important aim of the current investigation.

### Limitation

As a limitation of the current investigation, it should be stated that neither a randomized controlled trial was conducted, nor a cross-over protocol, nor a follow-up evaluation was included. This was due to the complex design and to the reason that most participants were already highly affected by their disease impairments (high EDSS).

Due to the great heterogeneity of participants, no group * time interaction effects could be determined. Still, based on significant within-group differences and effect sizes for mono-articular mobility (WL) and motor accuracy (SL) our main results of functional improvement, as described above, could be underpinned. In addition, we experienced that even small increases in motor function without significant side effects (i.e. fatigue, pain, dizziness or disease progression) are a great success in patients with severe MS. Nevertheless, the final statistical proof of the superiority of a WBV treatment is lacking.

Finally, we recognized that subjective perception during everyday life were most important for the participants, but were not recorded scientifically. Those included bad, such as perception of physical stress, as well as good experiences, such as walking without walking aids for first time in years. Future investigations should focus on those individualized experiences.

## Conclusion

Six weeks of WBV intercepted progressive degeneration of mono-articular mobility and enhanced fine motor accuracy in the ankle joint enabling greater performance for functional mobility. Those effects can be of relevance in everyday life activities and can enhance quality of life in MS patients. By counteracting MS-related motor impairments, especially the population of highly affected patients might benefit from an individualized WBV training. However, further investigations are still needed: While WBV training has been described to be time-efficient and easy-to-apply, it is crucially essential to provide individualized and flexible support during the training process to prevent any possible side-effects due to physical stress.

## Supporting information

S1 Fig Basic position during whole-body vibration training. (TIFF)

S1 Checklist CONSORT 2010 checklist of information to include when reporting a randomised trial*. (PDF)

S1 File (PDF)

S2 File (PDF)
