# Supplementary figures and images for: Six weeks of whole-body vibration improves fine motor accuracy, functional mobility and quality of life in people with multiple sclerosis
Source: PLoS One. 2022 Jul 11;17(7):e0270698. doi: 10.1371/journal.pone.0270698 (PMC9273076; doi:10.1371/journal.pone.0270698)

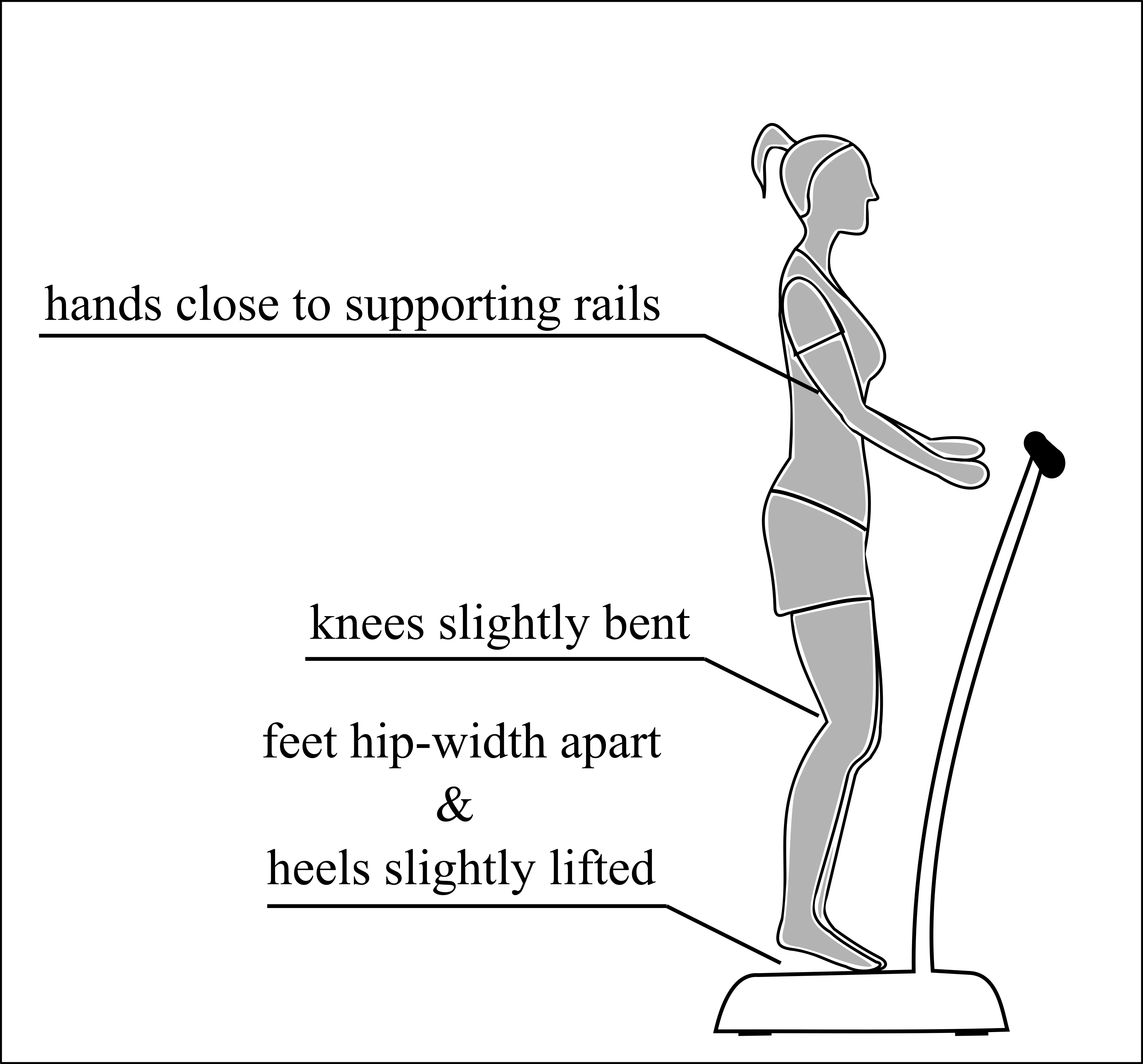

Supplement: S1 Fig — (TIFF) [file pone.0270698.s001.tiff]
