# Supplementary material for: Six weeks of whole-body vibration improves fine motor accuracy, functional mobility and quality of life in people with multiple sclerosis
Source: PLoS One. 2022 Jul 11;17(7):e0270698. doi: 10.1371/journal.pone.0270698 (PMC9273076; doi:10.1371/journal.pone.0270698)
Supplement: S1 File — (PDF) [file pone.0270698.s003.pdf]

Protocol

for the research project

Whole body vibration training as a therapeutic approach for multiple sclerosis - Effect on neuromuscular correlates in motor control and locomotion.

Submitted by

Dr. Ramona Ritzmann, Prof. Dr. Albert Gollhofer and Anne Krause

Institut für Sport und Sportwissenschaft der Albert-Ludwigs-Universität Freiburg

1. Scientific background

Multiple sclerosis (MS) is a neurodegenerative and progressively progressive autoimmune disease of the central nervous system. Inflammatory processes damage the protective sheaths of the nerves, which is accompanied by the loss of axons [1-4]. The clinical picture is diverse in its course, severity, and manifestation, with different symptoms depending on the structures affected. The main symptoms initially include sensory disturbances, visual disturbances, and disturbances in muscle function, which may manifest as acute weakness, paralysis, or increased muscle stiffness. In the course of the disease, these are often accompanied by chronic spasticity, muscle atrophy, and progressive loss of strength, resulting in gait disturbances and impaired stance control and unsteadiness in purposeful movements
[1, 5]. These symptoms are partly caused by the disease itself and partly due to the physical inactivity that accompanies it [6-9].

The latter, in particular, has been shown to be counteracted by physical activity [10]. Therefore, in the recent past, non-drug therapeutic approaches have become increasingly important to counteract the progressive degeneration in MS.

Empirical studies with endurance or strength training interventions [11] have already shown that MS patients with mild to moderate severity were able to improve both their muscle strength and functional mobility during activities of daily living through physical activity [7, 12, 13]. Furthermore, fatigue, well-being, and quality of life have been shown to be improved by physical activity [14]. Recent findings even discuss neuroprotective effects, i.e., a disease-modifying, anti-inflammatory effect through activity, so that a slowing of disease progression could be induced. In contrast, possible negative effects in relation to relapses and disease-related adverse events due to physical activity could be excluded [15].

In neurorehabilitation, whole-body vibration in particular has become the focus of research interest in the last decade as an effective training method. The neuromuscular system is highly challenged by the vibration of the sup port surface. Via reflex arcs, muscle spindles (receptors) and spinal excitation transmission are addressed via Ia afferents and α-motoneurons in the spinal cord [16, 17]. A highfrequency sequence of stretch reflexes results [5, 18-20], which has a stimulatory effect on sensorimotor interaction. This reflex muscle activation has positive effects on motor function and movement control [21, 22]. Studies document these improvements for both healthy [23-29] and neurologically diseased subject groups, such as stroke patients, people with polyneuropathy, or cerebral palsy [16, 30-35]. The applicability of the training to the clinical and home environment is

simple, as it does not require a sports therapy training area compared to conventional methods. However, in relation to its application in patients suffering from MS, the evidence regarding the effects of whole-body vibration training is limited: Only a few studies investigated acute [31, 36] as well as exercise-induced adaptations to whole-body vibration [37-44]. For example, Schuhfried et al [31] demonstrated that improved postural control and functional mobility can be achieved by a vibration session; however, some of the values just missed the significance level. Adjustments after multi-week training sessions (longitudinal studies over 3-20 weeks) varied between significant positive [38, 40, 41, 44] and small effects from whole-body vibration [37, 43]. Improvements from whole-body vibration include increased gait, or strength endurance [40, 41], increased leg muscle strength [38, 44], and improvements in functional aspects of postural control [41] and mobility [44]. However, while the aforementioned studies describe only functional adaptations to whole-body
vibration, evidence on the underlying neuromuscular mechanisms is widely lacking. Furthermore, the heterogeneity in the training devices used as well as the different designs complicate the comparability of the presented data [45, 46].

This leads to the question for the present investigation whether training with whole-body vibration can induce neuromuscular adaptations, which ultimately manifest themselves functionally in improved stance control and locomotion.

2. Rational and project collaborators

In the planned study, neuromuscular adaptations to a training intervention with whole-body vibration in patients with MS will be investigated and evaluated in terms of stance control and locomotion. For this purpose, postural control, functional mobility and capacity as well as fine motor skills of the subjects will be assessed in different measurement paradigms before and after a whole-body vibration training intervention. The research methodology is borrowed from biomechanics and neurophysiology. The present project is conducted in cooperation with the Institute of Exercise and Occupational Medicine of the University Medical Center Freiburg and Prof. Dr. Albert Gollhofer, Institute of Sports and Sports Science of the Albert-Ludwigs-University Freiburg under the direction of Dr. Ramona Ritzmann and Anne Krause.

3. Desing and methodolgy

The present study is a longitudinal investigation in which neuromuscular and functional correlates are examined before (t0), after (t1) and four weeks after (follow-up, t2) a four-week training with whole-body vibration.

The training sessions will be completed on a side-alternating vibration plate (Galileo®, Novotec Medical GmbH). The extent of the training sessions will be two sessions per week of 3x3

minutes each. Intensities will be set to a minimum of 26Hz at amplitudes of 2-4mm (depending on body size) following Jackson et al [36]. During the individualized training program [6], standardized baseline conditions are defined based on the study by Ritzmann et al [47].

The examination series is preceded by a screening for which the reliable and valid measuring instrument "Expanded Disability Status Scale, EDSS" [48] as well as the patient's medical letter are used. This serves the purpose of being able to make a suitable subject delimitation in advance [13]. Accordingly, patients with an EDSS score <7 and motor deficits in the lower extremity should be included. Exclusion criteria include at least one acute episode in the previous three months as well as acute injury and chronic diseases. In addition, the method of transcranial magnetic stimulation (TMS) should be used to diagnose reduced nerve conduction velocity [2], so that exclusion criteria also apply according to the guidelines for use with TMS [49].

- To elicit neuromuscular and functional correlates, four different measurement paradigms are used in the input (t0) and output measurements (t1 & t2), which include Balance tests to check postural control,
- Mobility tests (active mobility - maximum possible deflection of the ankle and knee joint),
- Functional capacity tests (testing rate of force increase, reactive strength, and jumping strength, times up and go test)
- Fine motor function tests (fine motor response to external signals) include.

During these tasks, the following measurement methods will be used: Electromyography (EMG) to collect muscle activity, peripheral nerve stimulation (PNS) to collect spinal excitability, joint goniometry (ankle, knee and hip joint) to collect joint angle changes and mobility, posturography (measurement of foot pressure point) to collect balance ability, and mechanography (force measurement) to collect force-related parameters. All of the measurement methods described are common diagnostic instruments, are available at the Institute of Sport and Sport Science and are backed up by an ethical vote of the Albert Ludwig University of Freiburg.

In addition to the methods mentioned above, adjustments of disease-related symptoms such as fatigue, pain and depression during the measurement time points will be checked with the help of questionnaires.

4. Potential - expected findings for the therapy of MS patients

The aim of the project is to assess the benefits and effectiveness of whole-body vibration training as a sports therapy measure for MS patients. The evidence of underlying neuromuscular mechanisms also offers the possibility to modify therapeutic measures in MS in order to apply an individual, disease-specific training. Based on the research presented above, it can be hypothesized that neuromuscular adaptations of whole-body vibration training have a positive effect on functional stance control and locomotion in MS patients.

Whole-body vibration offers above all the potential of a low-time training, in which passive highintensity training is performed on the vibration plate. The training can therefore be easily integrated into the clinical or home routine of the patients and allows to counteract inactivity and disease-related degeneration at the central and peripheral level.

5. Time line

- Subject recruitment: 6 weeks
- Preparation and test measurements: 4 weeks
- Initial measurement: 6 weeks
- Training phase (with time offset for capacity reasons): 14 weeks
- Baseline measurement: 6 weeks
- Data evaluation and statistical analysis: 12 weeks
- Final report: 4 weeks.
- The total duration of the study is therefore 12 months.

6. Contact

Dr. Ramona Ritzmann

Institut für Sport und Sportwissenschaft

Albert-Ludwigs-Universität Freiburg, Schwarzwaldstraße 175, 79117 Freiburg

Email: ramona.ritzmann@sport.uni-freiburg.de, Tel: 0761-203 4559

7. Literature

[1] Amatya B, Khan F, La Mantia L, Demetrios M,Wade DT (2013). Non pharmacological interventions for spasticity in multiple sclerosis Cochrane Database Syst Rev 2): CD009974.
[2] Compston A,Coles A (2002). Multiple sclerosis Lancet 359(9313): 1221–1231.
[3] Garner, Dena J P,Widrick JJ (2003). Cross-bridge mechanisms of muscle weakness in multiple sclerosis Muscle & nerve 27(4): 456–464.
[4] Noseworthy JH, Lucchinetti C, Rodriguez M,Weinshenker BG (2000). Multiple sclerosis The New England journal of medicine 343(13): 938–952.
[5] Cochrane DJ (2011). Vibration exercise: the potential benefits International journal of sports medicine 32(2): 75–99.
[6] Dalgas U, Stenager E,Ingemann-Hansen T (2008). Multiple sclerosis and physical exercise: recommendations for the application of resistance-, endurance- and combined training Multiple sclerosis (Houndmills, Basingstoke, England) 14(1): 35–53.
[7] Giesser BS (2015). Exercise in the management of persons with multiple sclerosis Therapeutic advances in neurological disorders 8(3): 123–130.
[8] Kent-Braun JA, Ng AV, Castro M, Weiner MW, Gelinas D, Dudley GA,Miller RG (1997). Strength, skeletal muscle

composition, and enzyme activity in multiple sclerosis Journal of applied physiology (Bethesda, Md. : 1985) 83(6): 1998–2004.
[9] Ng AV,Kent-Braun JA (1997). Quantitation of lower physical activity in persons with multiple sclerosis Medicine and science in sports and exercise 29(4): 517–523.
[10] Heesen C, Romberg A, Gold S,Schulz K (2006). Physical exercise in multiple sclerosis: supportive care or a putative diseasemodifying treatment Expert review of neurotherapeutics 6(3): 347–355.
[11] Sabapathy NM, Minahan CL, Turner GT,Broadley SA (2011). Comparing endurance- and resistance-exercise training in people with multiple sclerosis: a randomized pilot study Clinical rehabilitation 25(1): 14–24.
[12] Latimer-Cheung AE, Pilutti LA, Hicks AL, Martin Ginis, Kathleen A, Fenuta AM, MacKibbon KA,Motl RW (2013). Effects of exercise training on fitness, mobility, fatigue, and health-related quality of life among adults with multiple sclerosis: a systematic review to inform guideline development Archives of physical medicine and rehabilitation 94(9): 1800-1828.e3.
[13] Rietberg MB, Brooks D, Uitdehaag BM,Kwakkel G (2011). Exercise therapy for multiple sclerosis. Cochrane Database Syst Rev. 2005.
[14] Dalgas U, Stenager E, Jakobsen J, Petersen T, Hansen HJ, Knudsen C, Overgaard K,Ingemann-Hansen T (2010). Fatigue, mood and quality of life improve in MS patients after progressive resistance training Multiple sclerosis (Houndmills, Basingstoke, England) 16(4): 480–490.
[15] Pilutti LA, Platta ME, Motl RW,Latimer-Cheung AE (2014). The safety of exercise training in multiple sclerosis: a systematic review Journal of the neurological sciences 343(1-2): 3–7.
[16] Ahlborg L, Andersson C,Julin P (2006). Whole-body vibration training compared with resistance training: effect on spasticity, muscle strength and motor performance in adults with cerebral palsy Journal of rehabilitation medicine 38(5): 302–308.
[17] Cardinale M,Bosco C (2003). The use of vibration as an exercise intervention Exercise and sport sciences reviews 31(1): 3–7.
[18] Rittweger J, Mutschelknauss M,Felsenberg D (2003). Acute changes in neuromuscular excitability after exhaustive whole body vibration exercise as compared to exhaustion by squatting exercise Clin Physiol Funct Imaging 23(2): 81–86.
[19] Ritzmann R, Kramer A, Gruber M, Gollhofer A,Taube W (2010). EMG activity during whole body vibration: motion artifacts or stretch reflexes? Eur. J. Appl. Physiol. 110(1): 143–151.
[20] Ritzmann R, Kramer A, Gollhofer A,Taube W (2011). The effect of whole body vibration on the H-reflex, the stretch reflex, and the short-latency response during hopping Scand J Med Sci Sports.
[21] Ness LL,Field-Fote EC (2009). Effect of whole-body vibration on quadriceps spasticity in individuals with spastic hypertonia due to spinal cord injury Restor. Neurol. Neurosci. 27(6): 621–631.
[22] Stark C, Nikopoulou-Smyrni P, Stabrey A, Semler O,Schoenau E (2010). Effect of a new physiotherapy concept on bone mineral density, muscle force and gross motor function in children with bilateral cerebral palsy JOURNAL OF MUSCULOSKELETAL & NEURONAL INTERACTIONS 10(2): 151–158.
[23] Berschin G,Sommer HM (2004). Vibration strength training and joint stabilization: EMG based examination of the influence of vibration frequency and posture on muscle activation and co-activation DEUTSCHE ZEITSCHRIFT FUR SPORTMEDIZIN 55(6): 152–156.
[24] Bogaerts A, Delecluse C, Claessens AL, Coudyzer W, Boonen S,Verschueren, Sabine M P (2007). Impact of whole-body vibration training versus fitness training on muscle strength and muscle mass in older men: a 1-year randomized controlled trial J. Gerontol. A Biol. Sci. Med. Sci. 62(6): 630–635.
[25] Bogaerts A, Verschueren S, Delecluse C, Claessens AL,Boonen S (2007). Effects of whole body vibration training on postural control in older individuals: a 1 year randomized controlled trial Gait Posture 26(2): 309–316.
[26] Delecluse C, Roelants M,Verschueren S (2003). Strength increase after whole-body vibration compared with resistance training Med Sci Sports Exerc 35(6): 1033–1041.
[27] Gusi N, Raimundo A,Leal A (2006). Low-frequency vibratory exercise reduces the risk of bone fracture more than walking: a randomized controlled trial BMC Musculoskelet Disord 7): 92.
[28] Ritzmann R, Kramer A, Bernhardt S,Gollhofer A (2014). Whole body vibration training--improving balance control and muscle endurance PLoS ONE 9(2): e89905.
[29] Spiliopoulou SI, Amiridis IG, Tsigganos G,Hatzitaki V (2013). Side-alternating vibration training for balance and ankle muscle strength in untrained women J Athl Train 48(5): 590–600.
[30] Marín PJ, Ferrero CM, Menéndez H, Martín J,Herrero AJ (2013). Effects of whole-body vibration on muscle architecture, muscle strength, and balance in stroke patients: a randomized controlled trial Am J Phys Med Rehabil 92(10): 881–888.
[31] Schuhfried O, Mittermaier C, Jovanovic T, Pieber K,Paternostro-Sluga T (2005). Effects of whole-body vibration in patients with multiple sclerosis: a pilot study Clinical rehabilitation 19(8): 834–842.
[32] Tankisheva E, Bogaerts A, Boonen S, Feys H,Verschueren S (2014). Effects of intensive whole-body vibration training on muscle strength and balance in adults with chronic stroke: a randomized controlled pilot study Arch Phys Med Rehabil 95(3): 439–446.
[33] Tihanyi J, Di Giminiani R, Tihanyi T, Gyulai G, Trzaskoma L,Horváth M (2010). Low resonance frequency vibration affects strength of paretic and non-paretic leg differently in patients with stroke Acta Physiol Hung 97(2): 172–182.
[34] Tihanyi TK, Horváth M, Fazekas G, Hortobágyi T,Tihanyi J (2007). One session of whole body vibration increases voluntary muscle strength transiently in patients with stroke Clin Rehabil 21(9): 782–793.
[35] Turbanski S, Haas CT, Schmidtbleicher D, Friedrich A,Duisberg P (2005). Effects of random whole-body vibration on postural control in Parkinson's disease Res Sports Med 13(3): 243–256.
[36] Jackson KJ, Merriman HL, Vanderburgh PM,Brahler CJ (2008). Acute effects of whole-body vibration on lower extremity muscle performance in persons with multiple sclerosis Journal of neurologic physical therapy : JNPT 32(4): 171–176.
[37] Broekmans T, Roelants M, Alders G, Feys P, Thijs H,Eijnde BO (2010). Exploring the effects of a 20-week whole-body vibration training programme on leg muscle performance and function in persons with multiple sclerosis J Rehabil Med 42(9): 866–872.
[38] Claerbout M, Gebara B, Ilsbroukx S, Verschueren S, Peers K, van Asch P,Feys P (2012). Effects of 3 weeks' whole body

vibration training on muscle strength and functional mobility in hospitalized persons with multiple sclerosis Mult Scler 18(4): 498–505.
[39] Eftekhari E, Mostahfezian M, Etemadifar M,Zafari A (2012). Resistance training and vibration improve muscle strength and functional capacity in female patients with multiple sclerosis Asian J Sports Med 3(4): 279–284.
[40] Hilgers C, Mundermann A, Riehle H,Dettmers C (2013). Effects of whole-body vibration training on physical function in patients with multiple sclerosis NeuroRehabilitation 32(3): 655–663.
[41] Mason RR, Cochrane DJ, Denny GJ, Firth EC,Stannard SR (2012). Is 8 weeks of side-alternating whole-body vibration a safe and acceptable modality to improve functional performance in multiple sclerosis? Disabil Rehabil 34(8): 647–654.
[42] Schyns F, Paul L, Finlay K, Ferguson C,Noble E (2009). Vibration therapy in multiple sclerosis: a pilot study exploring ist effects on tone, muscle force, sensation and functional performance Clin Rehabil 23(9): 771–781.
[43] Wolfsegger T, Assar H,Topakian R (2014). 3-week whole body vibration does not improve gait function in mildly affected multiple sclerosis patients--a randomized controlled trial J Neurol Sci 347(1-2): 119–123.
[44] Wunderer K, Schabrun SM,Chipchase LS (2010). Effects of whole body vibration on strength and functional mobility in multiple sclerosis Physiother Theory Pract 26(6): 374–384.
[45] del Pozo-Cruz B, Adsuar JC, Parraca JA, del Pozo-Cruz J, Olivares PR,Gusi N (2012). Using whole-body vibration training in patients affected with common neurological diseases: a systematic literature review J Altern Complement Med 18(1): 29–41.
[46] Santos-Filho SD, Cameron MH,Bernardo-Filho M (2012). Benefits of whole-body vibration with an oscillating platform for people with multiple sclerosis: a systematic review Mult Scler Int 2012): 274728.
[47] Ritzmann R, Gollhofer A,Kramer A (2013). The influence of vibration type, frequency, body position and additional load on the neuromuscular activity during whole body vibration European journal of applied physiology 113(1): 1–11.
[48] Kurtzke JF (1983). Rating neurologic impairment in multiple sclerosis: an expanded disability status scale (EDSS) Neurology 33(11): 1444–1452.
[49] Keel JC, Smith MJ,Wassermann EM (2001). A safety screening questionnaire for transcranial magnetic stimulation Clinical Neurophysiology 112(4): 720.
