# Supplementary material for: Six weeks of whole-body vibration improves fine motor accuracy, functional mobility and quality of life in people with multiple sclerosis
Source: PLoS One. 2022 Jul 11;17(7):e0270698. doi: 10.1371/journal.pone.0270698 (PMC9273076; doi:10.1371/journal.pone.0270698)
Supplement: S2 File — (PDF) [file pone.0270698.s004.pdf]

Protokoll

zum Forschungsvorhaben

Ganzkörpervibrationstraining als Therapieansatz bei Multipler Sklerose Effekt auf neuromuskuläre Korrelate bei der Motorischen Kontrolle und Lokomotion

eingereicht von

Dr. Ramona Ritzmann, Prof. Dr. Albert Gollhofer und Anne Krause

Institut für Sport und Sportwissenschaft der Albert-Ludwigs-Universität Freiburg

1. Wissenschaftlicher Hintergrund

Multiple Sklerose (MS) ist eine neurodegenerative und progressiv fortschreitende Autoimmunerkrankung des zentralen Nervensystems. Durch Entzündungsprozesse werden die Schutzhüllen der Nerven geschädigt, was mit dem Verlust von Axonen einhergeht [1–4]. Das Krankheitsbild ist im Verlauf, im Schweregrad und in der Ausprägung vielfältig und weist je nach betroffenen Strukturen unterschiedliche Symptome auf. Zu den Hauptsymptomen zählen zunächst Empfindungsstörungen, Sehstörungen und Störungen der Muskelfunktion, welche sich als akute Kraftlosigkeit, Lähmung oder erhöhte Muskelsteifigkeit zeigen können. Diese gehen im Verlauf der Erkrankung häufig mit chronischer Spastik, Muskelatrophie sowie progressivem Kraftverlust und dadurch verursachten Gangstörungen und Beeinträchtigungen bei der Standkontrolle sowie

Unsicherheit bei zielgerichteten Bewegungen einher [1, 5]. Diese Symptome sind zum einen durch die Krankheit selbst verursacht und zum anderen der mit ihr einhergehenden körperlichen Inaktivität geschuldet [6–9].

Insbesondere letztgenannter kann nachweislich durch körperliche Betätigung entgegengewirkt werden [10]. Daher gewannen in der jüngsten Vergangenheit zunehmend nicht-medikamentöse Therapieansätze an Bedeutung, um der fortschreitenden Degeneration bei MS entgegen zu wirken.

Durch empirische Untersuchungen mit Ausdauer- oder Krafttrainingsinterventionen [11] konnte bereits aufgezeigt werden, dass MS-Patienten mit mildem bis moderatem Schweregrad durch körperliche Aktivität sowohl ihre Muskelkraft als auch die funktionelle Mobilität bei alltagsrelevanten Aktivitäten verbessern konnten [7, 12, 13]. Ferner werden Fatigue, Wohlbefinden und Lebensqualität nachweislich durch körperliche Aktivität verbessert [14]. Neueste Erkenntnisse diskutieren sogar neuroprotektive Effekte, also eine krankheits-modifizierende, entzündungshemmende Wirkung durch Aktivität, sodass eine Verlangsamung des Krankheitsverlaufes induziert werden könnte. Mögliche negative Effekte in Bezug zu Rückfällen und krankheitsbezogenen unerwünschten Ereignissen durch körperliche Aktivität konnten hingegen ausgeschlossen werden [15].

In der Neurorehabilitation ist im letzten Jahrzehnt insbesondere die Ganzkörpervibration als wirkungsvolle Trainingsmethode in den Fokus des Forschungsinteresses gerückt. Durch die Vibration der Unterstützungsfläche wird das neuromuskuläre System in hohem Maße gefordert. Über Reflexbögen werden die Muskelspindeln (Rezeptoren) und die spinale Erregungsweiterleitung über Ia-Afferenzen und α-Motoneurone im Rückenmark angesprochen [16, 17]. Es resultiert eine hochfrequente Abfolge von Dehnreflexen [5, 18–20], was

stimulierend auf die sensomotorische Interaktion wirkt. Diese reflektorische Muskelaktivierung hat positive Effekte auf die Motorik und Bewegungskontrolle [21, 22]. Studien dokumentieren diese Verbesserungen sowohl für gesunde [23–29] als auch für neurologisch erkrankte Probandengruppen, wie etwa Schlaganfallpatienten, Menschen mit Polyneuropathie oder Zerebralparese [16, 30–35]. Die Umsetzbarkeit des Trainings ins klinische und häusliche Umfeld ist einfach, da es im Vergleich zu herkömmlichen Methoden keiner sporttherapeutischen Trainingsfläche bedarf.

In Bezug zur Anwendung bei Patienten, die an MS leiden, ist die Evidenz bezüglich der Effekte von Ganzkörpervibrationstraining jedoch begrenzt: Nur wenige Studien untersuchten akute [31, 36] sowie trainings-induzierte Anpassungen an Ganzkörpervibration [37–44]. So konnten Schuhfried et al. [31] aufzeigen, dass eine verbesserte Posturale Kontrolle und funktionelle Mobilität durch eine Vibrationseinheit erzielt werden kann; die Werte verfehlten teilweise jedoch knapp das Signifikanzniveau. Anpassungen nach mehr-wöchigen Trainingseinheiten (Längsschnittuntersuchen über 3-20 Wochen) variieren zwischen signifikant positiven [38, 40, 41, 44] und geringen Effekten durch Ganzkörpervibration [37, 43]. Die Verbesserungen durch Ganzkörpervibration umfassen eine gesteigerte Gang-, bzw. Kraftausdauer [40, 41], eine erhöhte Muskelkraft der Beinmuskulatur [38, 44] sowie Verbesserungen funktioneller Aspekte der Posturalen Kontrolle [41] und Mobilität [44]. Während die genannten Studien jedoch ausschließlich funktionelle Anpassungen an Ganzkörpervibration beschreiben, fehlen weitläufig Nachweise über die zugrundeliegenden neuromuskulären Mechanismen. Des Weiteren erschweren die Heterogenität bei den verwendeten Trainingsgeräten sowie die unterschiedlichen Designs die Vergleichbarkeit der dargestellten Angaben [45, 46].

Daraus ergibt sich die Fragestellung für vorliegende Untersuchung, ob durch ein Training mit Ganzkörpervibration neuromuskuläre Anpassungen hervorgerufen werden können, welche sich schließlich funktionell in einer verbesserten Standkontrolle und Lokomotion äußern.

2. Forschungsidee und Projektpartner

In der geplanten Untersuchung werden neuromuskuläre Anpassungen an eine Trainingsintervention mit Ganzkörpervibration bei Patienten mit MS untersucht und auf die Standkontrolle und Lokomotion übertragen evaluiert. Hierfür werden in unterschiedlichen Messparadigmen die Posturale Kontrolle, die funktionelle Mobilität und Kapazität sowie die Feinmotorik der Probanden vor und nach einer Ganzkörpervibration-Trainingsintervention erhoben. Die Forschungsmethodik ist der Biomechanik und Neurophysiologie entlehnt. Das vorliegende Projekt wird in einer Kooperation dem Institut für Bewegungs- und Arbeitsmedizin des Universitätsklinikums Freiburg und Prof. Dr. Albert Gollhofer, Institut für Sport und

Sportwissenschaft der Albert-Ludwigs-Universität Freiburg unter der Leitung von Frau Dr. Ramona Ritzmann und Anne Krause durchgeführt.

3. Studiendesign und Methoden

Bei der vorliegenden Studie handelt es sich um eine Längsschnittuntersuchung, bei der neuromuskuläre und funktionelle Korrelate vor (t_0), nach (t_1) und vier Wochen nach (follow-up, t_2) einem vier-wöchigen Training mit Ganzkörpervibration untersucht werden.

Die Trainingseinheiten werden auf einer seit-alternierenden Vibrationsplatte (Galileo®, Novotec Medical GmbH) absolviert. Der Umfang der Trainingseinheiten wird sich auf zwei Einheiten pro Woche à 3x3 Minuten belaufen. Intensitäten werden in Anlehnung an Jackson et al. [36] auf mindestens 26Hz bei Amplituden von 2-4mm (je nach Körpergröße) festgelegt. Während des individualisierten Trainingsprogramms [6] werden standardisierte Ausgangsbedingungen auf Grundlage der Untersuchung von Ritzmann et al. [47] definiert.

Der Untersuchungsreihe wird ein Screening voran gestellt, für welches das reliable und valide Messinstrument „Expanded Disability Status Scale, EDSS“ [48] sowie der Arztbrief des Patienten verwendet werden. Dies dient dazu, bereits im Voraus eine geeignete Probandeneingrenzung vornehmen zu können [13]. Demnach sollen Patienten mit EDSS-Wert <7 sowie motorischen Defiziten in der unteren Extremität eingeschlossen werden. Ausschlusskriterien umfassen hingegen mind. einen akuten Schub in den vorangegangenen drei Monaten sowie akute Verletzung und chronische Erkrankungen. Zusätzlich dazu soll die Methode der Transkraniellen Magnetstimulation (TMS) zur Diagnose reduzierter Nervenleitgeschwindigkeit herangezogen werden [2], sodass ebenfalls Ausschlusskriterien nach den Richtlinien für die Anwendung mit TMS gelten [49].

Zur Erhebung neuromuskulärer und funktioneller Korrelate werden in den Ein- (t_0) und Ausgangsmessungen (t_1 & t_2) vier unterschiedliche Messparadigmen verwendet, welche

- Gleichgewichtstests zur Überprüfung der Posturalen Kontrolle,
- Mobilitätstests (aktive Beweglichkeit - maximal mögliche Auslenkung des Sprung- und Kniegelenks),
- Tests zur funktionellen Kapazität (Überprüfung der Kraftanstiegsrate, der reaktiven Kraft sowie der Sprungkraft, Times Up and Go Test)
- Tests zur Feinmotorik (feinmotorische Reaktion auf externe Signale) beinhalten.

Während dieser Aufgaben werden nachfolgende Messmethoden genutzt: Elektromyographie (EMG) zur Erhebung der Muskelaktivität, periphere Nervenstimulation (PNS) zur Erhebung der spinalen Erregbarkeit, Gelenksgoniometrie (Sprung-, Knie- und Hüftgelenk) zur Erhebung der Gelenkwinkelveränderungen und -mobilität, Posturographie (Messung des Fuß-Druck-

Punktes) zur Erhebung der Gleichgewichtsfähigkeit sowie Mechanographie (Kraftmessung) zur Erhebung kraftbezogener Kenngrößen. Alle dargestellten Messverfahren sind geläufige Diagnostik-Instrumente, stehen am Institut für Sport und Sportwissenschaft zur Verfügung und werden über ein Ethikvotum der Albert-Ludwigs-Universität Freiburg abgesichert.
In Ergänzung zu den genannten Methoden werden zudem Anpassungen krankheitsbedingter Symptome wie Fatigue, Schmerz und Depression während den Messzeitpunkten mithilfe von Fragebögen überprüft.

4. Potential - Zu erwartende Erkenntnis für die Therapie von MS Patienten

Das Vorhaben dient dazu, Nutzen und Wirkweise des Ganzkörpervibrationstrainings als sporttherapeutische Maßnahme für MS Patienten abzuschätzen zu können. Der Nachweis zugrundeliegender neuromuskulärer Mechanismen bietet zudem die Möglichkeit, therapeutische Maßnahmen bei MS zu modifizieren, um so ein individuelles, krankheitsspezifisches Training zu applizieren. Auf Basis des oben dargestellten Forschungsstandes kann angenommen werden, dass sich neuromuskuläre Anpassungen des Ganzkörpervibrationstrainings positiv auf die funktionelle Standkontrolle und Lokomotion bei MS-Patienten auswirkt. Ganzkörpervibration bietet vor allen Dingen das Potential eines zeitarmen Trainings, bei welchem passiv hoch-intensiv auf der Vibrationsplatte trainiert wird. Das Training kann demnach einfach in den klinischen oder häuslichen Alltag der Patienten integriert werden und ermöglicht, der Inaktivität und krankheitsbedingten Degeneration auf zentraler und peripherer Ebene entgegen zu wirken.

5. Zeitplanung

- Probandenrekrutierung: 6 Wochen
- Vorbereitung und Testmessungen: 4 Wochen
- Eingangsmessung: 6 Wochen
- Trainingsphase (aus Kapazitätsgründen mit zeitlichem Versatz): 14 Wochen
- Ausgangsmessung: 6 Wochen
- Datenauswertung und statistische Analyse: 12 Wochen
- Abschlussbericht: 4 Wochen.
- Die Gesamtlaufzeit der Studie beträgt demnach 12 Monate.

6. Kontakt:

Dr. Ramona Ritzmann
Institut für Sport und Sportwissenschaft
Albert-Ludwigs-Universität Freiburg, Schwarzwaldstraße 175, 79117 Freiburg

Email: ramona.ritzmann@sport.uni-freiburg.de, Tel: 0761-203 4559

7. Literatur

[1] Amatya B, Khan F, La Mantia L, Demetrios M,Wade DT (2013). Non pharmacological interventions for spasticity in multiple sclerosis Cochrane Database Syst Rev 2): CD009974.
[2] Compston A,Coles A (2002). Multiple sclerosis Lancet 359(9313): 1221–1231.
[3] Garner, Dena J P,Widrick JJ (2003). Cross-bridge mechanisms of muscle weakness in multiple sclerosis Muscle & nerve 27(4): 456–464.
[4] Noseworthy JH, Lucchinetti C, Rodriguez M,Weinshenker BG (2000). Multiple sclerosis The New England journal of medicine 343(13): 938–952.
[5] Cochrane DJ (2011). Vibration exercise: the potential benefits International journal of sports medicine 32(2): 75–99.
[6] Dalgas U, Stenager E,Ingemann-Hansen T (2008). Multiple sclerosis and physical exercise: recommendations for the application of resistance-, endurance- and combined training Multiple sclerosis (Houndmills, Basingstoke, England) 14(1): 35–53.
[7] Giesser BS (2015). Exercise in the management of persons with multiple sclerosis Therapeutic advances in neurological disorders 8(3): 123–130.
[8] Kent-Braun JA, Ng AV, Castro M, Weiner MW, Gelinas D, Dudley GA,Miller RG (1997). Strength, skeletal muscle composition, and enzyme activity in multiple sclerosis Journal of applied physiology (Bethesda, Md. : 1985) 83(6): 1998–2004.
[9] Ng AV,Kent-Braun JA (1997). Quantitation of lower physical activity in persons with multiple sclerosis Medicine and science in sports and exercise 29(4): 517–523.
[10] Heesen C, Romberg A, Gold S,Schulz K (2006). Physical exercise in multiple sclerosis: supportive care or a putative diseasemodifying treatment Expert review of neurotherapeutics 6(3): 347–355.
[11] Sabapathy NM, Minahan CL, Turner GT,Broadley SA (2011). Comparing endurance- and resistance-exercise training in people with multiple sclerosis: a randomized pilot study Clinical rehabilitation 25(1): 14–24.
[12] Latimer-Cheung AE, Pilutti LA, Hicks AL, Martin Ginis, Kathleen A, Fenuta AM, MacKibbon KA,Motl RW (2013). Effects of exercise training on fitness, mobility, fatigue, and health-related quality of life among adults with multiple sclerosis: a systematic review to inform guideline development Archives of physical medicine and rehabilitation 94(9): 1800-1828.e3.
[13] Rietberg MB, Brooks D, Uitdehaag BM,Kwakkel G (2011). Exercise therapy for multiple sclerosis. Cochrane Database Syst Rev. 2005.
[14] Dalgas U, Stenager E, Jakobsen J, Petersen T, Hansen HJ, Knudsen C, Overgaard K,Ingemann-Hansen T (2010). Fatigue, mood and quality of life improve in MS patients after progressive resistance training Multiple sclerosis (Houndmills, Basingstoke, England) 16(4): 480–490.
[15] Pilutti LA, Platta ME, Motl RW,Latimer-Cheung AE (2014). The safety of exercise training in multiple sclerosis: a systematic review Journal of the neurological sciences 343(1-2): 3–7.
[16] Ahlborg L, Andersson C,Julin P (2006). Whole-body vibration training compared with resistance training: effect on spasticity, muscle strength and motor performance in adults with cerebral palsy Journal of rehabilitation medicine 38(5): 302–308.
[17] Cardinale M,Bosco C (2003). The use of vibration as an exercise intervention Exercise and sport sciences reviews 31(1): 3–7.
[18] Rittweger J, Mutschelknauss M,Felsenberg D (2003). Acute changes in neuromuscular excitability after exhaustive whole body vibration exercise as compared to exhaustion by squatting exercise Clin Physiol Funct Imaging 23(2): 81–86.
[19] Ritzmann R, Kramer A, Gruber M, Gollhofer A,Taube W (2010). EMG activity during whole body vibration: motion artifacts or stretch reflexes? Eur. J. Appl. Physiol. 110(1): 143–151.
[20] Ritzmann R, Kramer A, Gollhofer A,Taube W (2011). The effect of whole body vibration on the H-reflex, the stretch reflex, and the short-latency response during hopping Scand J Med Sci Sports.
[21] Ness LL,Field-Fote EC (2009). Effect of whole-body vibration on quadriceps spasticity in individuals with spastic hypertonia due to spinal cord injury Restor. Neurol. Neurosci. 27(6): 621–631.
[22] Stark C, Nikopoulou-Smyrni P, Stabrey A, Semler O,Schoenau E (2010). Effect of a new physiotherapy concept on bone mineral density, muscle force and gross motor function in children with bilateral cerebral palsy JOURNAL OF MUSCULOSKELETAL & NEURONAL INTERACTIONS 10(2): 151–158.
[23] Berschin G,Sommer HM (2004). Vibration strength training and joint stabilization: EMG based examination of the influence of vibration frequency and posture on muscle activation and co-activation DEUTSCHE ZEITSCHRIFT FUR SPORTMEDIZIN 55(6): 152–156.
[24] Bogaerts A, Delecluse C, Claessens AL, Coudyzer W, Boonen S,Verschueren, Sabine M P (2007). Impact of whole-body vibration training versus fitness training on muscle strength and muscle mass in older men: a 1-year randomized controlled trial J. Gerontol. A Biol. Sci. Med. Sci. 62(6): 630–635.
[25] Bogaerts A, Verschueren S, Delecluse C, Claessens AL,Boonen S (2007). Effects of whole body vibration training on postural control in older individuals: a 1 year randomized controlled trial Gait Posture 26(2): 309–316.
[26] Delecluse C, Roelants M,Verschueren S (2003). Strength increase after whole-body vibration compared with resistance training Med Sci Sports Exerc 35(6): 1033–1041.
[27] Gusi N, Raimundo A,Leal A (2006). Low-frequency vibratory exercise reduces the risk of bone fracture more than walking: a randomized controlled trial BMC Musculoskelet Disord 7): 92.
[28] Ritzmann R, Kramer A, Bernhardt S,Gollhofer A (2014). Whole body vibration training--improving balance control and muscle endurance PLoS ONE 9(2): e89905.
[29] Spiliopoulou SI, Amiridis IG, Tsigganos G,Hatzitaki V (2013). Side-alternating vibration training for balance and ankle muscle strength in untrained women J Athl Train 48(5): 590–600.

[30] Marín PJ, Ferrero CM, Menéndez H, Martín J,Herrero AJ (2013). Effects of whole-body vibration on muscle architecture, muscle strength, and balance in stroke patients: a randomized controlled trial Am J Phys Med Rehabil 92(10): 881–888.
[31] Schuhfried O, Mittermaier C, Jovanovic T, Pieber K,Paternostro-Sluga T (2005). Effects of whole-body vibration in patients with multiple sclerosis: a pilot study Clinical rehabilitation 19(8): 834–842.
[32] Tankisheva E, Bogaerts A, Boonen S, Feys H,Verschueren S (2014). Effects of intensive whole-body vibration training on muscle strength and balance in adults with chronic stroke: a randomized controlled pilot study Arch Phys Med Rehabil 95(3): 439–446.
[33] Tihanyi J, Di Giminiani R, Tihanyi T, Gyulai G, Trzaskoma L,Horváth M (2010). Low resonance frequency vibration affects strength of paretic and non-paretic leg differently in patients with stroke Acta Physiol Hung 97(2): 172–182.
[34] Tihanyi TK, Horváth M, Fazekas G, Hortobágyi T,Tihanyi J (2007). One session of whole body vibration increases voluntary muscle strength transiently in patients with stroke Clin Rehabil 21(9): 782–793.
[35] Turbanski S, Haas CT, Schmidtbleicher D, Friedrich A,Duisberg P (2005). Effects of random whole-body vibration on postural control in Parkinson's disease Res Sports Med 13(3): 243–256.
[36] Jackson KJ, Merriman HL, Vanderburgh PM,Brahler CJ (2008). Acute effects of whole-body vibration on lower extremity muscle performance in persons with multiple sclerosis Journal of neurologic physical therapy : JNPT 32(4): 171–176.
[37] Broekmans T, Roelants M, Alders G, Feys P, Thijs H,Eijnde BO (2010). Exploring the effects of a 20-week whole-body vibration training programme on leg muscle performance and function in persons with multiple sclerosis J Rehabil Med 42(9): 866–872.
[38] Claerbout M, Gebara B, Ilsbroukx S, Verschueren S, Peers K, van Asch P,Feys P (2012). Effects of 3 weeks' whole body vibration training on muscle strength and functional mobility in hospitalized persons with multiple sclerosis Mult Scler 18(4): 498–505.
[39] Eftekhari E, Mostahfezian M, Etemadifar M,Zafari A (2012). Resistance training and vibration improve muscle strength and functional capacity in female patients with multiple sclerosis Asian J Sports Med 3(4): 279–284.
[40] Hilgers C, Mundermann A, Riehle H,Dettmers C (2013). Effects of whole-body vibration training on physical function in patients with multiple sclerosis NeuroRehabilitation 32(3): 655–663.
[41] Mason RR, Cochrane DJ, Denny GJ, Firth EC,Stannard SR (2012). Is 8 weeks of side-alternating whole-body vibration a safe and acceptable modality to improve functional performance in multiple sclerosis? Disabil Rehabil 34(8): 647–654.
[42] Schyns F, Paul L, Finlay K, Ferguson C,Noble E (2009). Vibration therapy in multiple sclerosis: a pilot study exploring ist effects on tone, muscle force, sensation and functional performance Clin Rehabil 23(9): 771–781.
[43] Wolfsegger T, Assar H,Topakian R (2014). 3-week whole body vibration does not improve gait function in mildly affected multiple sclerosis patients--a randomized controlled trial J Neurol Sci 347(1-2): 119–123.
[44] Wunderer K, Schabrun SM,Chipchase LS (2010). Effects of whole body vibration on strength and functional mobility in multiple sclerosis Physiother Theory Pract 26(6): 374–384.
[45] del Pozo-Cruz B, Adsuar JC, Parraca JA, del Pozo-Cruz J, Olivares PR,Gusi N (2012). Using whole-body vibration training in patients affected with common neurological diseases: a systematic literature review J Altern Complement Med 18(1): 29–41.
[46] Santos-Filho SD, Cameron MH,Bernardo-Filho M (2012). Benefits of whole-body vibration with an oscillating platform for people with multiple sclerosis: a systematic review Mult Scler Int 2012): 274728.
[47] Ritzmann R, Gollhofer A,Kramer A (2013). The influence of vibration type, frequency, body position and additional load on the neuromuscular activity during whole body vibration European journal of applied physiology 113(1): 1–11.
[48] Kurtzke JF (1983). Rating neurologic impairment in multiple sclerosis: an expanded disability status scale (EDSS) Neurology 33(11): 1444–1452.
[49] Keel JC, Smith MJ,Wassermann EM (2001). A safety screening questionnaire for transcranial magnetic stimulation Clinical Neurophysiology 112(4): 720.
